# Molecular dissection of transcriptional reprogramming of steviol glycosides synthesis in leaf tissue during developmental phase transitions in *Stevia rebaudiana* Bert

**DOI:** 10.1038/s41598-017-12025-y

**Published:** 2017-09-19

**Authors:** Gopal Singh, Gagandeep Singh, Pradeep Singh, Rajni Parmar, Navgeet Paul, Radhika Vashist, Mohit Kumar Swarnkar, Ashok Kumar, Sanatsujat Singh, Anil Kumar Singh, Sanjay Kumar, Ram Kumar Sharma

**Affiliations:** 1 Biotechnology Department, CSIR-Institute of Himalayan Bioresource Technology, Palampur, Himachal Pradesh India; 2 Academy of Scientific and Innovative Research, New Delhi, India; 3 Agrotechnology of Medicinal, Aromatic and Commercially Important Plants, CSIR-Institute of Himalayan Bioresource Technology, Palampur, Himachal Pradesh India; 4 ICAR-Indian Institute of Agricultural Biotechnology, PDU Campus, IINRG, Namkum, Ranchi, Jharkhand India

## Abstract

Stevia is a natural source of commercially important steviol glycosides (SGs), which share biosynthesis route with gibberellic acids (GAs) through plastidal MEP and cytosolic MVA pathways. Ontogeny-dependent deviation in SGs biosynthesis is one of the key factor for global cultivation of Stevia, has not been studied at transcriptional level. To dissect underlying molecular mechanism, we followed a global transcriptome sequencing approach and generated more than 100 million reads. Annotation of 41,262 *de novo* assembled transcripts identified all the genes required for SGs and GAs biosynthesis. Differential gene expression and quantitative analysis of important pathway genes (DXS, HMGR, KA13H) and gene regulators (WRKY, MYB, NAC TFs) indicated developmental phase dependent utilization of metabolic flux between SGs and GAs synthesis. Further, identification of 124 CYPs and 45 UGTs enrich the genomic resources, and their PPI network analysis with SGs/GAs biosynthesis proteins identifies putative candidates involved in metabolic changes, as supported by their developmental phase-dependent expression. These putative targets can expedite molecular breeding and genetic engineering efforts to enhance SGs content, biomass and yield. Futuristically, the generated dataset will be a useful resource for development of functional molecular markers for diversity characterization, genome mapping and evolutionary studies in Stevia.

## Introduction

Plants are rich and vital source of a large variety of pharmaceutically and industrially important natural metabolites^1^. *Stevia rebaudiana* Bert. (Stevia, family-Asteraceae), is a shrubby, perennial plant species^2^, popular worldwide for its ability to accumulate considerably high level of several commercially important steviol glycosides (SGs; up to ~20% of total dry weight)^3,4^. These SGs have been used as an alternative natural sweetener and are effective for controlling important modern lifestyle diseases (diabetes, obesity, cardiac blockage and hypertension)^5,6^. Based on carbohydrate moiety and its position, SGs have been classified as Steviosides, Rebaudiosides (A–E), Dulcosides, Steviobiosides, and Rubusosides^3,7^. Being ~300 times sweeter than sucrose with low glycemic index, Stevioside and Rebaudioside-A are among the commercially most popular SGs^8^. Therefore, despite being native to South America (Paraguay, Argentina and Brazil), Stevia cultivation has been expanded globally to China, Japan, Australia, Canada, USA and India^2,9^. In India, it is mainly cultivated in Rajasthan, Kerala, Maharashtra, Orissa and Himachal Pradesh, and has been expanded to the other parts of the country^10^.

SGs synthesis utilize the combined metabolic flux of cytosolic mevalonic acid (MVA) and plastidal methyl erythritol 4-phosphate (MEP) pathways^11^ (Figure S1). Geranylgeranyl pyrophosphate (C-20), the common precursor for the synthesis of all diterpenoids, is produced by geranylgeranyl pyrophosphate synthase (GGPPS) after condensing four isoprene units. The introduction of ent-cyclization by ent-copalyl pyrophosphate synthase (CPPS) specify the metabolic flux towards ent-diterpenoids such as steviol glycosides. Involvement of ent-kaurane synthase (KS) and ent-kaurane oxidase (KO) leads to the synthesis of ent-kaurenoic acid^3,11^. This ent-kaurenoic acid is the last shared intermediate of SGs and gibberellic acids (GAs) biosynthesis. The action of two different ER-membrane located cytochrome P450 monooxygenases (CYPs): ent-kaurenoic acid hydroxylase (KA13H) and ent-kaurenoic acid oxidase (KAO), results in the formation of steviol and GA12, respectively^12^. Further, cytosolic glycosylation of steviol by four cytosolic UDP-glucosyltransferases (UGTs) gives rise to different types of steviol glycosides, while, GA12 acts as a precursor for the synthesis of all kinds of GAs^13^. The shared synthesis route with GAs and involvement of multiple cellular compartments makes SGs biosynthesis a more complex process.

Several physiological and phytochemical studies indicate the higher accumulation of SGs in vegetative phase till appearance of floral bud followed by a declining in flowering phase^14,15^. Although, change in SGs content has been highlighted in previous studies, nonetheless, global molecular mechanism to identify key candidates that influence SGs accumulation during different phases of plant development have not been elucidated, so far. Thus, it becomes imperative to understand developmental phase dependent expression pattern of the genes involed in SGs biosynthesis, and to identify other putative key candidates. *De novo* transcriptome sequencing using various NGS platforms have emerged as a robust, efficient and cost-effective approach to understand genome-wide expression patterns in non-reference plants^16,17^. In the current study, global transcriptome sequencing approach was adopted to understand the effect of developmental phase transitions on the expression of the genes required for SGs biosynthesis. Further, efforts were also made to identify and classify putative candidates such as CYPs and UGTs that assist the modification and diversification of secondary metabolism. Further, Protein-protein interactome (PPI) network analysis of CYPs and UGTs with genes involved in SGs biosynthesis was performed to identify the presence of putative hub proteins that may directly or indirectly regulate the SGs accumulation. The current study will provide a comprehensive genomic resource for manipulating SGs accumulation through genetic engineering, and implementation of molecular breeding approaches for dissection of major agronomic traits and varietal improvement programs in Stevia.

## Results

### Illumina sequencing and *de novo* assembly

**Figure 1:**
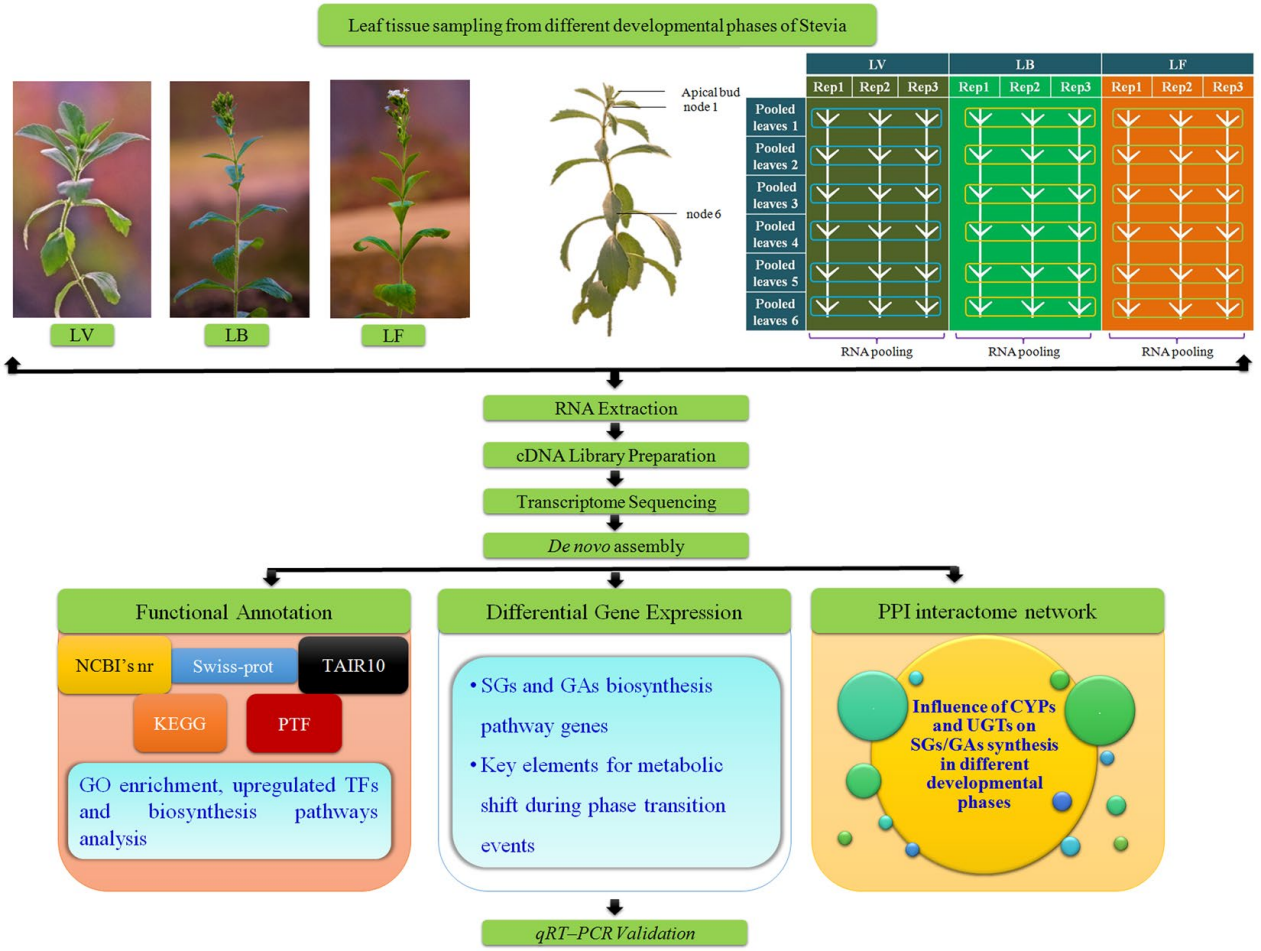
Schematic representation of methodology adopted for comparative leaf transcriptome analysis and function annotation during developmental phase transition. Leaf tissues from each respective node of three biological replicates were pooled together, hence, total six leaf tissues were used for RNA isolation for each developmental phase. Equimolar concentration from six RNA samples was used for library preparation. Abbreviations are as follows: LV (leaf tissues in vegetative phase), LB (leaf tissues in bud phase), LF (leaf tissues in flowering phase), SGs (steviol glycosides), GAs (gibberellic acids), CYPs (cytochrome P450 monooxygenases), and UGTs (UDP-glucosyltransferases).

To study gene expression pattern in the leaf tissues during developmental phase transitions in Stevia (Figure 1), three cDNA libraries (LV: leaf tissue in vegetative phase, LB: leaf tissue in bud phase, and LF: leaf tissue in flowering phase) were sequenced using Illumina GAIIx platform. After quality assessment and data filtering (removal of low quality, contaminated reads and adaptor sequences), 17,055,744, 14,299,157 and 17,610,069 filtered reads were obtained for LV, LB and LF, respectively. Further, to improve the *de novo* assembly and downstream annotations, in-house (unpublished) high quality filtered reads of young floral bud (B; 18,300,946) and fully bloomed flower tissues (F; 15,027,649) were also included (Table 1). Out of 101.6 million raw reads, a total 82,293,555 filtered reads were *de novo* assembled into 41,262 transcripts with average length, N_50_ and CG content of 922 bases, 1,244 bases and 39.3%, respectively (Figure S2, Table S1). To validate the quality of *de novo* assembly, mapping of high quality filtered reads to the assembled transcripts resulted a high alignment rate of 86.82% (71,340,904 mapped reads, Table 1). Secondly, alignments of publicly available EST sequences of Stevia (5,548) obtained mapping rate of 95.71%. The raw reads of Illumina sequencing for all the samples have been deposited in National Centre for Biotechnology Information (NCBI) Sequence Read Archive (SRA) with accession number SRP094030 under BioProject PRJNA355055.

**Table 1:** Characteristics distribution of different types of reads (raw, filtered and mapped) obtained in Illumina sequencing.

Samples	Total raw reads	Total filtered reads	% of filtered reads	Total mapped reads	% of mapped reads	Multiple mapped reads	% of multiple mapped reads
LV	21154108	17055744	80.63	14740654	86.43	69521	0.4
LB	17908854	14299157	79.84	12746727	89.14	62209	0.4
LF	21234036	17610069	82.93	15069021	85.57	81620	0.5
B	22711170	18300946	80.58	15926339	85.21	77548	0.5
F	18592680	15027649	80.83	13189957	87.77	63830	0.4
Total	**101600848**	**82293565**	**80.96**	**71340904**	**86.82**	**354728**	**0.4**

### Functional annotation and classification of assembled transcripts

**Figure 2:**
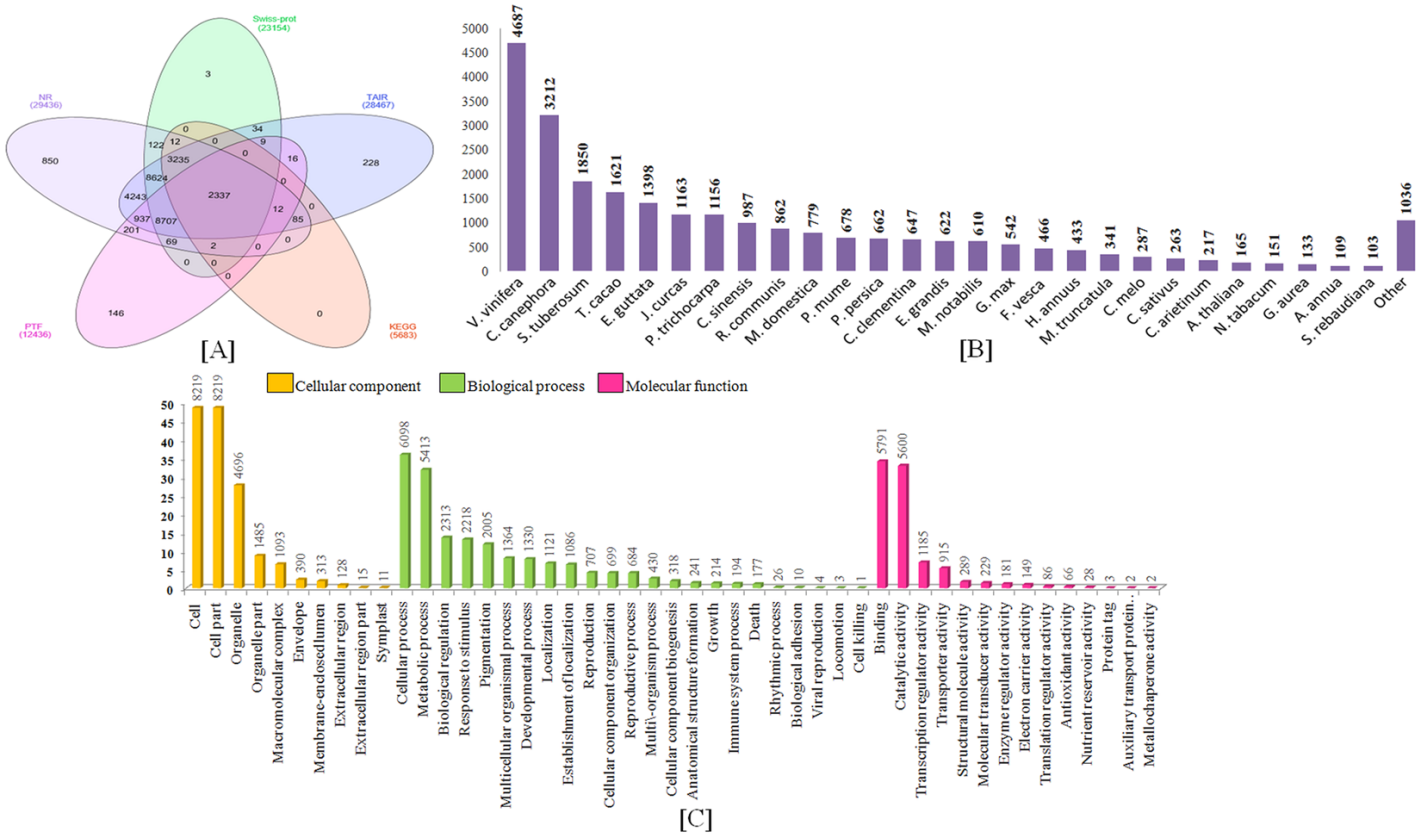
Summary of functional annotation of Stevia transcripts. (**A**) Venn diagram representing the abundance of annotations with five different protein databases, (**B**) Histogram representing the species wise homology distribution of Stevia sequences in NCBI’s nr protein database annotations. (**C**) Histogram representing three broad categories, Cellular components, Molecular function; and Biological process. X-axis elucidating different GO categories, Y-axis (left) indicating the percentage of annotation to each GO category, Y-axis (right) depicted the scale for GO terms in a single GO category.

To obtain the comprehensive functional insights of assembled transcripts, five main databases (NCBI’s non-redundant, Swiss-Prot, TAIR 10, KEGG and PTF) were used to search homologs of Stevia transcripts using the BLASTx. Out of the total 41,262 transcripts, 29,436 (71.34%), 28,467 (70.0%), 23,154 (56.11%), 8,888 (21.54%) and 5,683 (13.77%) were annotated against NCBI’s nr, TAIR10, Swiss-Prot, PTF and KEGG database, respectively, with 2337 transcripts common in all annotations (Figure 2A). This also revealed highest homology with some of the well-explored plant systems like *Vitis vinifera* (15.92%), *Coffea canephora* (10.91%), *Solanum tuberosum* (6.28%), *Theobroma cacao* (5.50%), *Erythranthe guttata* (4.75%), *Jatropha curcas* (3.95%) and *Populus trichocarpa* (3.92%). However, due to limited genomic information, only 103 transcripts were annotated with *Stevia rebaudiana* entries in NCBI’s nr database **(**Figure 2B **)**.

Gene Ontology (GO) has been widely used to assign functional terms to uncharacterized sequences obtained by transcriptome sequencing^18^. A total of 16,853 transcripts were successfully assigned to 65,751 GO terms (47 functional groups), in which 26,656 (40%) classified into 23 categories of biological processes, 24,569 (37%) into 10 functional categories of cellular component, and 14,526 (27%) into 14 functional categories of molecular function (Figure 2C). More interpretations revealed that the ‘cellular process’ (GO:0009987) in biological processes, ‘cell part’ (GO:0044464) in cellular component and ‘binding’ (GO:0005488) in molecular function were found to be the most abundant functional groups with 8,219, 6,098 and 5,791 GO counts, respectively. For cellular component, a high proportion of annotations were given to ‘cell’ (GO:0005623) and ‘organelle’ (GO:0043226); while ‘metabolic process’ (GO:0008152) and ‘response to stimulus’ (GO:0050896) were more abundant in biological process. Interestingly, categories which specify the events related to plant development and phase transition like ‘developmental process’ (GO:0032502), ‘reproduction’ (GO:0000003), and ‘reproductive process’ (GO:0022414), were also found significantly represented (Figure 2C).

Annotation with KEGG database can facilitate the biological function of the genes/pathway distributions^19^. A total of 5,683 transcripts revealed the significant match with default statistical parameters, and were assigned to 332 different KEGG pathways. Of these, metabolic pathways (819 hits), biosynthesis of secondary metabolites (352), photosynthesis (44), starch and sucrose metabolism pathway (38), plant hormone signal transduction (37) and carbon fixation (24), were the most enriched pathways (Table S2). The KEGG pathway analysis also identified 23 pathways representing gene network for secondary metabolites biosynthesis (Table 2). Of these, terpenoid backbone synthesis (30 hits), diterpenoid biosynthesis (8 hits) and monoterpenoid biosynthesis (3 hits) were reportedly involved in SGs biosynthesis. In order to identify the enzymes actively involved in various biological pathways in Stevia, the assembled transcripts were assigned their respective EC number by mapping against the KEGG database. Among them, members of Transferases class with 1563 hits were the most abundant followed by Oxidoreductases (903), Hydroxylases (770), Lyases (326), Ligases (262) and Isomerases (192) (Figure S3).

**Table 2:** Details of pathways involved in plant secondary metabolites synthesis revealed in KEGG database annotation.

S. No.	Pathway Id	Pathway description	Number of hits	Number of transcripts
1.	ko00900	Terpenoid backbone synthesis	30	48
2.	ko00130	Ubiquinone and other terpenoid-quinone biosynthesis	20	30
3.	ko00940	Phenylpropanoid biosynthesis	18	65
4.	ko00100	Steroid biosynthesis	18	23
5.	ko00906	Carotenoid biosynthesis	17	23
6.	ko00640	Propanoate metabolism	14	20
7.	ko00941	Flavonoid biosynthesis	9	13
8.	ko00960	Tropane, piperidine and pyridine alkaloid biosynthesis	9	15
9.	ko00950	Isoquinoline alkaloid biosynthesis	8	13
10.	ko00904	Diterpenoid biosynthesis	8	9
11.	ko00905	Brassinosteroid biosynthesis	5	6
12.	ko00945	Stilbenoid, diarylheptanoid and gingerol biosynthesis	5	20
13.	ko00909	Sesquiterpenoid and triterpenoid biosynthesis	5	5
14.	ko00945	Stilbenoid, diarylheptanoid and gingerol biosynthesis	5	20
15.	ko00908	Zeatin biosynthesis	4	4
16.	ko00902	Monoterpenoid biosynthesis	3	5
17.	ko00232	Caffeine metabolism	2	3
18.	ko00944	Flavone and flavonol biosynthesis	2	2
19.	ko00966	Glucosinolate biosynthesis	2	3
20.	ko00903	Limonine and pinene degradation	2	3
21.	ko00523	Polyketide sugar unit biosynthesis	2	2
22.	ko00942	Anthocyanin biosynthesis	1	1
23.	ko00281	Geraniol degradation	1	2

### Identification of the transcription factors encoding transcripts

**Figure 3:**
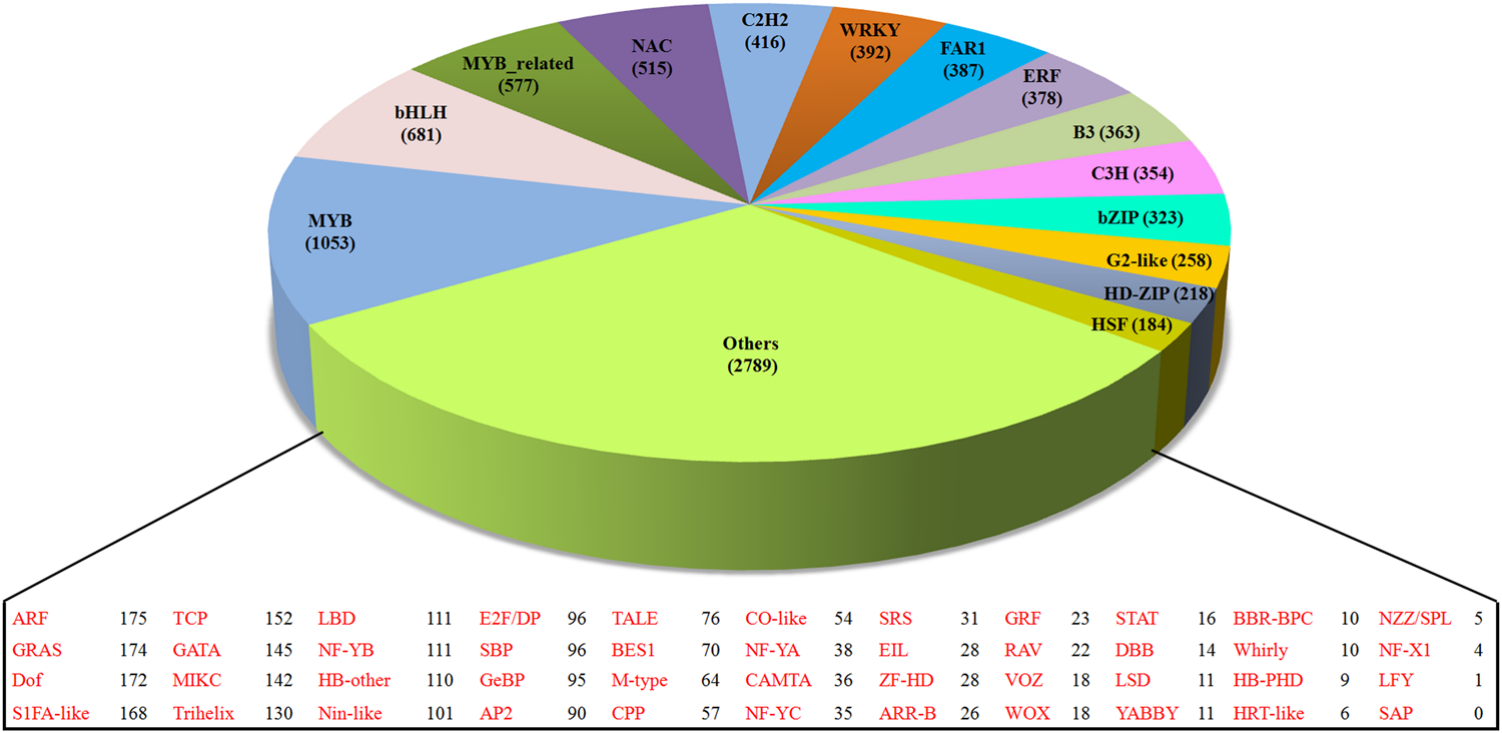
Pie-chart is representing the details and abundance of transcripts encoding different plant transcription factor family.

Transcription factors are the key gene regulators that control the expression of their targeted genes in eukaryotes through binding at respective promoter region^20^. For understanding the role of different transcription factors in plant metabolism, Stevia transcripts were annotated with Plant Transcription Factor (PTF) database with e-value of 10^−5^. A total of 8,888 transcripts were found to harbor the transcription factor domains which were further classified into 58 transcription factor families. Among these, MYB family (1,053) was the most represented, followed by bHLH (681), MYB-related (577) and NAC (515) families (Figure 3).

### Identification and classification of CYPs and UGTs

Recent studies illustrated the role of CYPs and UGTs in diversification of plant secondary metabolisms consequent to specific requirements of plant^21,22^. A total 307 transcripts exhibited homology against 124 different CYP proteins in Swiss-Prot annotation, including KO (CYP 701A3), KAO (CYP 88A3) and KA13H, well-known CYPs reportedly involved in SGs/GAs synthesis. Considering, KO was annotated with two homologs [(*Arabidopsis thaliana* ent-kaurene oxidase (Q93ZB2) and *Oryza sativa* ent-kaurene oxidase 2 (Q5Z5R4)], while KAO and KA13H were annotated against O23051 and Q0NZP1, respectively (Table S3). Furthermore, a total 118 transcripts were showing annotation for 45 UGTs including all three known UGTs of SGs biosynthesis with Q6VAB0, Q6VAA6 and Q6VAB4 for UDP 85C2, 74G1 and 76G1, respectively (Table S4).

### Global gene expression dynamics of leaf tissues during developmental phase transitions

Understanding the differential gene expression in tissue-specific manner is a common practice to identify and analyze the important genes/gene networks. For this, the filtered reads from three different RNA-Seq libraries were separately mapped to the assembled transcripts and were further normalized as RPKM (number of reads per kilobase per million mapped reads). Based on RPKM values, global gene expression of LV, LB and LF leaf tissues were classified into four different categories: the transcripts with RPKM value <2 (low expression), the transcripts with RPKM value 2.1–20 (moderate expression), the transcripts with RPKM value 20.1–100 (high expression), and the transcripts with RPKM value >100 (very high expression) (Figure S4A–C). Transcripts with a high level of expression were maximum in LB (5,978) followed by LF (5,863) and LV (5,622), while transcripts with very high expression were maximum for LF (1,395) followed by LB (1,313) and LV (1,244). The pair-wise differential gene expression analysis with edgeR statistics revealed 4,274, 5,380 and 3,498 transcripts were found differentially expressed in LV *vs* LB, LV *vs* LF, and LB *vs* LF combinations, respectively (Figure S4D–F). Interestingly, comparison of LV *vs* LF resulted in a discrete alteration in the DGEs as 3,456 transcripts were up-regulated in LV while such transcripts were only 1,824 in LF. Similarly, in case of LB *vs* LF, a total 2,637 transcripts were up-regulated in LB, while only 861 transcripts showed higher expression in LF tissue (Table S5).

### Identification of genes involved in steviol glycosides biosynthesis pathway

**Figure 4:**
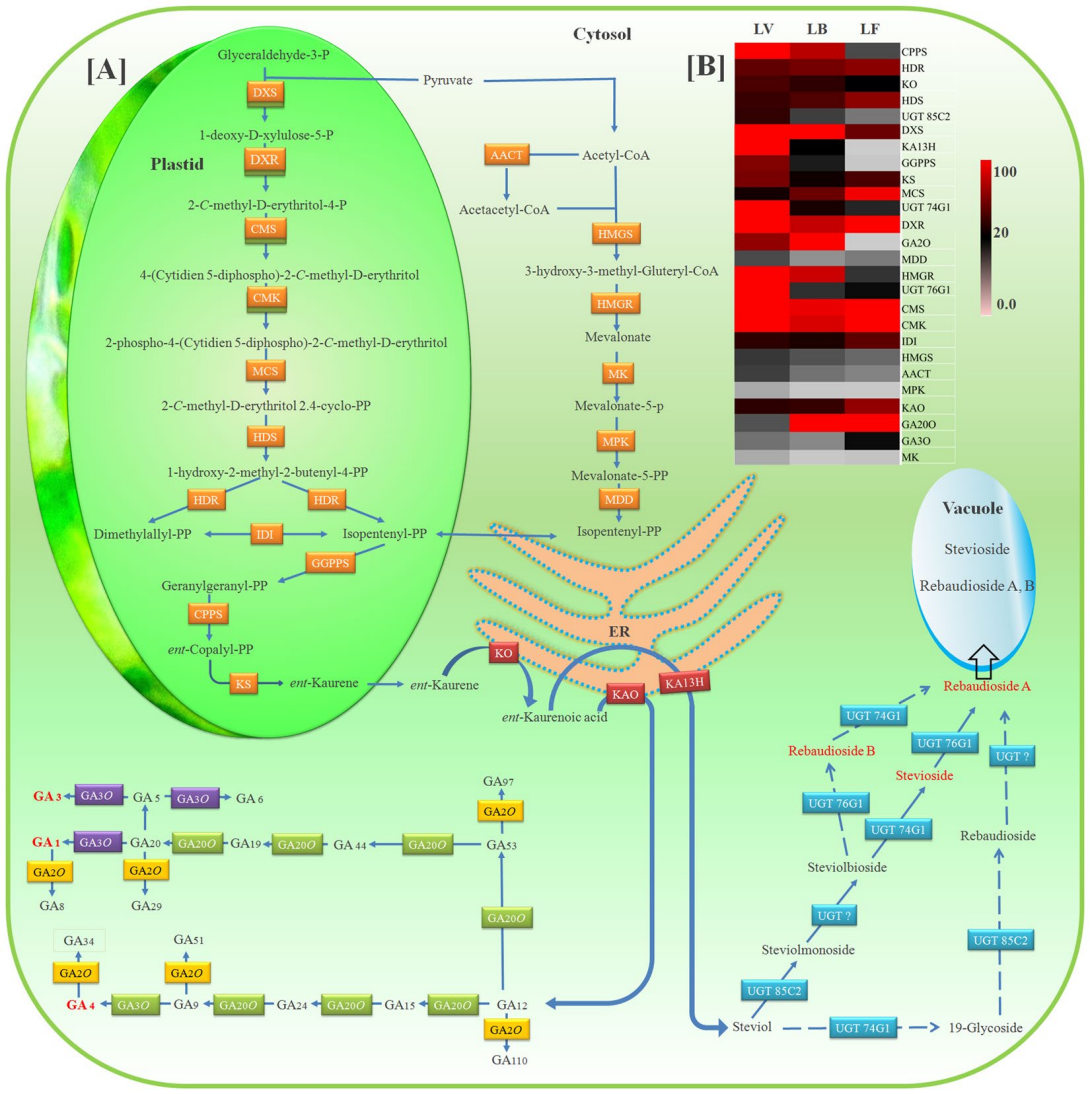
Diagrammatic representation and differential expression pattern of gene(s) involved in SGs/GAs biosynthesis. (**A**) MVA (Cytosolic) and MEP (Plastid)pathways is representing all the genes (Table S7), Dotted arrows depicting bioconversions reported *in vitro *only^13^, (**B**) Heatmap representing differential gene expression patterns of these genes in different leaf tissues during developmental phase transitions. Abbreviations are as follows: DXS (1-deoxy-D-xylulose-5-phosphate synthase), DXR (1-deoxy-D-xylulose 5-phosphate reductoisomerase), CMS (2-C-methyl-D-erythritol 4-phosphate cytidylyltransferase), CMK (4-diphosphocytidyl-2-C-methyl-D-erythritol kinase), MCS (2-C-methyl-D-erythritol 2,4-cyclodiphosphate synthase), HDS (4-hydroxy-3-methylbut-2-enyl diphosphate synthase), HDR (4-hydroxy-3-methylbut-2-enyl diphosphate reductase), AACT (acetyl Co-A acetyltransferase), HMGS (HMG-CoA synthase), HMGR (HMG-CoA reductase), MK (mevalonate kinase), MPK (phosphomevalonate kinase), MDD (diphosphomevalonate decarboxylase), IDI (isopentenyl-diphosphate delta-isomerase), GGPPS (geranylgeranyl pyrophosphate synthase), CPPS (ent-copalylpyrophosphate synthase), KS (ent-copalyl diphosphate synthase), KO (ent-kaurene oxidase), KA13H (ent-kaurenoic acid 13-hydroxylase), UGT 85C2 (UDP-glycosyltransferase 85C2), UGT 74G1 (UDP-glycosyltransferase 74G1), UGT 76G1 (UDP-glycosyltransferase 76G1), UGT? (unknown UGT), KAO (ent-kaurenoic acid oxidase), GA 20-O (gibberellin 20 oxidase), GA 3-O (gibberellin 3 oxidase), and GA 2-O (gibberellin 2 oxidase).

SGs are diterpenoid derivatives and share the biosynthesis route with GAs (Figure 4A). The precursor isoprene unit (5-C) is contributed by the bi-directional cross-talk between two well-characterized pathways: plastidal MEP and cytosolic MVA pathway^11^. Annotations of assembled transcripts facilitated the identification of all the genes for these two basic pathways (Table S6). The isoprene unit (IPP/DMAPP) is polymerized into the diterpene precursor (GGPP) with the help of a plastidal enzyme GGPPS. Further, ent-cyclization, a process unique to SGs/GAs synthesis is performed by CPPS enzyme. The catalytic action of KS followed by hydroxylation with ER-membrane located CYP protein- KO, results in the synthesis of ent-kaurenoic acid^3^. These two crucial enzymes of ent-diterpenoid biosynthesis were also successfully identified in the present study. Ent-kaurenoic acid is the last common intermediate in SGs and GAs synthesis and the introduction of hydroxyl (–OH) group at a different position by the action of two different CYPs segregate this into two different precursor molecules. KA13H, a member of CYPs protein family located at the ER membrane introduces –OH group at 13C position to form precursor skeleton for SGs synthesis known as “steviol”. While, the addition of an-OH group at 7^th^ position resulted into the synthesis of GA12 that act as a precursor for all gibberellins (Figure 4A). Further, Steviol undergoes the process of glycosylation performed by four UGTs to produce an array of SGs. However, GA12 is processed by different types of oxidases to produce different bioactive GAs (GA1, GA3 and GA4). Except for one unknown UGT, all the other genes participate in SGs and GAs biosynthesis were identified in our data (Table S6).

**Figure 5:**
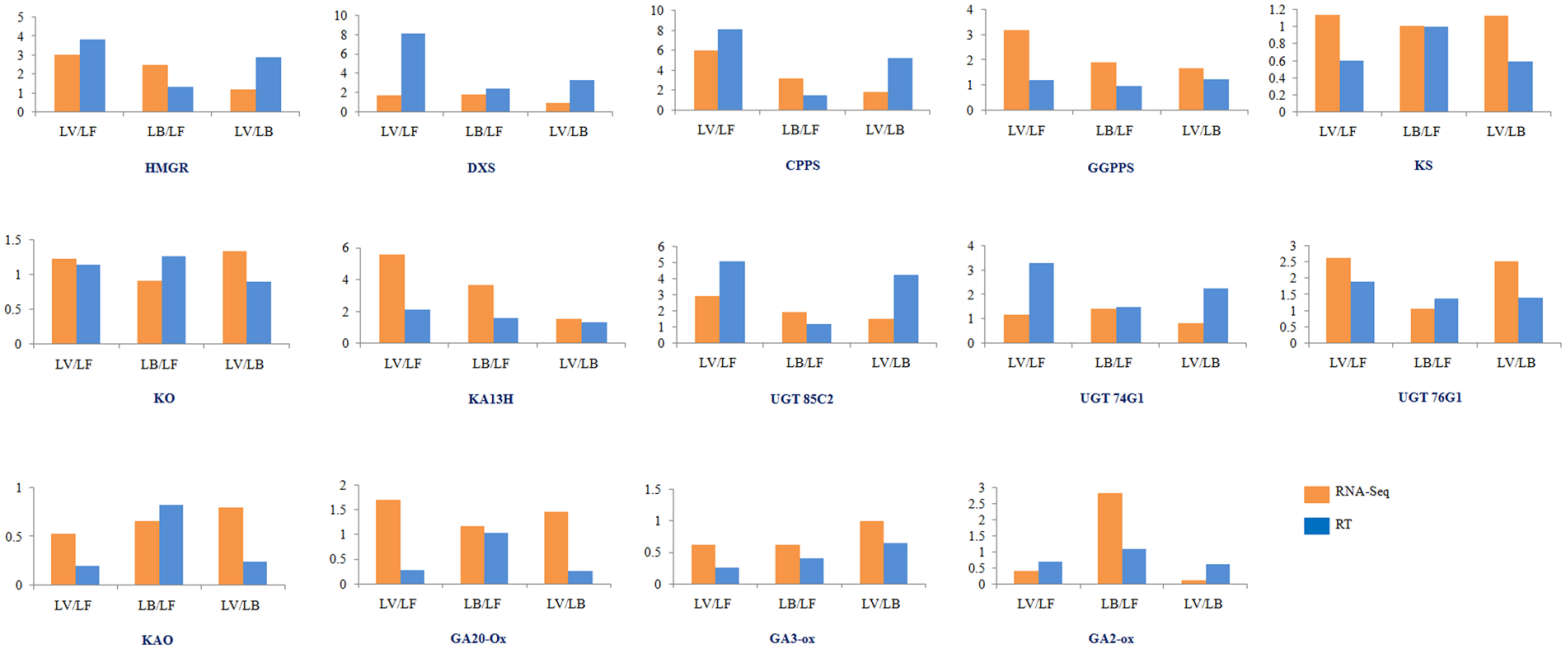
Histograms representing the comparative expression ratios obtained from RNA-seq data and qRT-PCR of key genes involved in SGs/GAs biosynthesis in LV, LB and LF tissues. The X-axis represents different combination of three leaf tissues for comparative expression analysis, Y-axis represents the fold change in RNA-seq and qRT-PCR analysis.

The comparative expression analysis revealed the abundance of transcripts encoding the enzymes, involved in SGs and GAs biosynthesis and were found to be differentially expressed. The rate limiting enzymes, such as HMGR (MVA pathway) and DXS (MEP pathway), were more expressed in LV compared to LB and LF tissues, while other genes showed relatively similar expression pattern during developmental phase transitions. The genes for ent-kaurenoic acid synthesis (KS and KO) showed similar expression, while expression of CPPS was higher in LV. The expression of SGs synthesis specific genes (KA13H, UGT 85C2, UGT 74G1 and UGT 76G1) was comparatively higher in LV as compared to LB and LF tissues. However, genes related to GAs biosynthesis (KAO, GA20O and GA3O) were expressed more in LF as compare to LV and LB tissues (Figure 4B). To validate the transcriptome data, 14 genes specifically required for SGs and GAs biosynthesis were selected for qRT-PCR analysis. The relative expression of these selected genes was calculated using GAPDH as an endogenous reference gene. qRT-PCR analysis also depicted the similar gene expression patterns as found in RNA-Seq data. The GGPPS was equally expressed while HMGR and CPPS were more in LV as compared to LB and LF tissues. KA13H and three UGTs were more expressed in LV followed by a decreasing trend along with the plant’s maturation (in LB and LF leaf tissues), whereas genes for GAs biosynthesis were more expressed in LB and LF tissues as compared to LV (Figure 5).

### Protein-protein interactome network analysis

**Figure 6:**
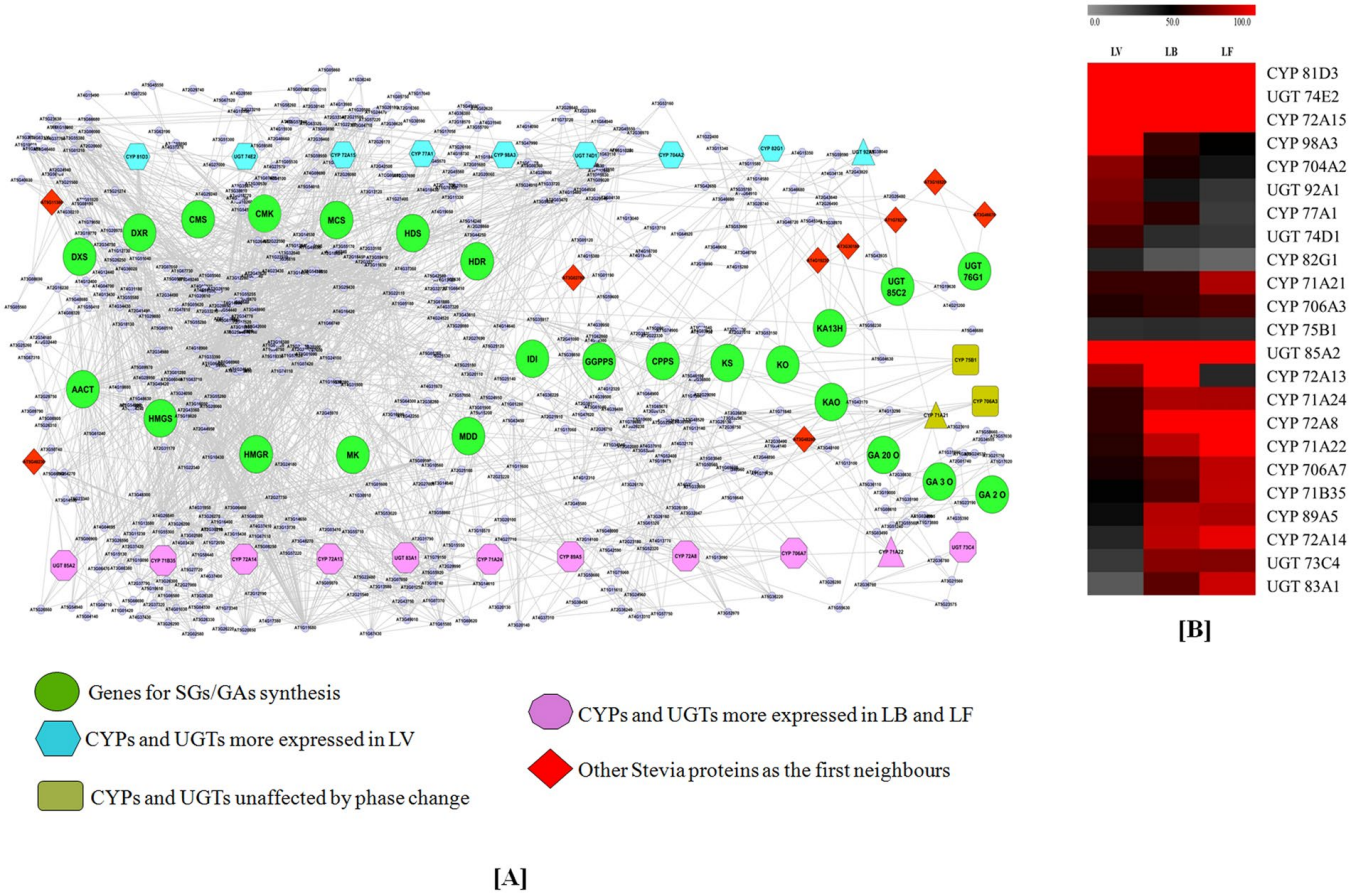
Protein-protein interactome (PPI) network analysis (**A**) CYPs and UGTs interactions with genes involved in SGs/GAs biosynthesis genes, (**B**) Heatmap representation of CYPs and UGTs with ≥5 interactions and differentially expressed in LV, LV and LF.

PPI network have become an effective approach for understanding the complex processes and solving many biological problems such as signaling, pathway identification and prediction of protein functions, and relationships between various kinds of proteins with different functions^23^. Considering the role of CYPs and UGTs in plant metabolic diversification^24^, 507 transcripts (including CYPs, UGTs and proteins involved in SGs/GAs biosynthesis) represented by 488 TAIR IDs were utilized for PPI network analysis to understand the influence of these diversifying proteins on SGs biosynthesis (Figure 6A). Network analysis revealed that 183 Stevia orthologs were interacting with 637 nodes having 2153 edges (with average numbers of neighbors: 6.760; clustering coefficient: 0.484) (Table S7). Interestingly, we found that AACT and HMGS (the initial enzymes of MVA pathway) were interacting with 64 and 25 neighbors, respectively. DXS (the first enzyme of MEP pathway) was found to interact with 8 other proteins in interactome network. GGPPS, an initial enzyme of SGs/GAs specifying diterpenoid biosynthesis was connected to 9 other proteins and GA20O, GA3O and GA2O were interacting with 1, 5 and 3 neighbors, respectively. The CYP 701A3 (KO) was showing interaction with 4 other proteins. KA13H, specify ent-kaurenoic acid to SGs biosynthesis was interacting with 6 other proteins. CYP 88A3 (KAO) shifts flux towards GAs synthesis, was connected to 8 neighbors including three other CYPs expressing in Stevia (CYP 71A21, CYP 71A22 and CYP 71A25). Interestingly, only two out of three known UGTs involve in SGs biosynthesis were identified in our network analysis. UGT 85C2 was interacting with three (AT1G78270, AT5G12890, AT4G34138) while, UGT 76G1was interacting with two neighbors (AT3G16520 AT3G46670). Absence of UGT 74G1 in the major interactome network may be due to the uniqueness of this UGT to Stevia.

Further, the CYPs and UGTs having ≥5 neighbours (putative hub proteins) in PPI network analysis and having higher expression (RPKM >20) were analyzed to find their influences on SGs/GAs biosynthesis during developmental phase transitions (Table S8). We found several CYPs (81D3, 72A15, 98A3, 704 A2, 98A3, 77A1 and 82G1) and UGTs (74E2, 92A1 and 74D1) showing significant interactions and higher expression in LV as compare to LB and LF. Similarly, many CYPs (72A3, 71A22, 71A24, 72A8, 706A7, 71B35, 89A5 and 72A14) and UGTs (85A2, 73C4 and 83A1) were highly expressed in LB and LF as compared to LV tissues (Table S8). UGT 92A1 was interacting with UGT 85C2 of SGs synthesis and was also expressed more in LV tissue. Moreover, CYP 71A22 was interacting with KAO (the CYP protein that shifts metabolic flux towards GAs synthesis) and showed higher expression in LB and LF (Figure 6B).

## Discussion

The genetic resources have been extensively explored for plant systems that are the source of many bioactive metabolites used in pharmaceutical, nutraceutical and flavor industries^25^. Considering multiple advantages of NGS sequencing to unravel the molecular/regulatory networks involved in developmental phase transitions^16,26–28^, *de novo* transcriptome sequencing approach was adopted to illustrate the mechanisms involve in altering SGs content in leaf tissues during vegetative, budding and flowering phases.

A total of 5.92 Gb transcriptome data and *de novo* assembly statistics (41,262 transcripts with an average length of 922 bases, N50; 1,244 bases) used to determine the efficiency of transcriptome sequencing, and was found to be comparable with earlier studies in Stevia^29,30^. The transcripts length (269–12,230 bases) with the abundance of >1,200 bases long transcripts (8,895 transcripts) (Figure S2), signify the presence of complete transcripts in our data. Furthermore, higher percentage of EST mapping (95.71%) and mapped reads (86.82%) to assembled transcripts significantly validate the high quality of *de novo* assembly^31^. Functional annotations of 71.34% transcripts with NCBI’s nr protein database suggest that a larger part of the data was annotated in this study. Nonetheless, 11,826 transcripts could not find homology in NCBI’s nr database, owing to the lack of Stevia specific protein information. Gene Ontology (GO) analysis showed that the abundance of transcripts involved in cellular process, binding and catalytic process categories, including other processes participated in plant developmental events. In KEGG annotation, a total of 23 pathways were found to be actively involved in secondary metabolites synthesis including pathways of SGs biosynthesis (Table 2), complemented the fact that about 15–25% of a plant genome is engaged for encoding proteins/enzymes involved in natural and secondary metabolite biosynthesis pathways^32^. Transcription factors are the key gene regulators to control gene expression during developmental phase transitions^20,33^. Predominant expression of members of MYB, bHLH, MYB-related and NAC families in Stevia expressome suggests their vital role in regulating secondary metabolism, cellular morphogenesis and plant growth regulators responsive signaling pathways^34,35^. Except for few small RNAs based studies^36,37^, no transcription factor family expansion has reported in Stevia. However, the members of bHLH (basic helix–loop–helix) family were reported to participate in phytochrome signaling during vegetative to reproductive phase transition in *Arabidopsis* ^38^. Similarly, MYC2 regulators (bHLH family) are known to be involved in many plant defense mechanisms in *Nicotiana attenuate* ^39^, while many WRKY proteins were involved in secondary metabolism^40^, biotic and abiotic stress tolerance^41^, trichome development and senescence^42^. Likewise, the regulatory role of NAC proteins was also documented in various developmental processes, defense, and abiotic stress responses^43^.

Both SGs and GAs, follow the common biosynthesis route by consuming 5C unit (IPP/DMAPP) contributed by MVA (cytosolic) and MEP (plastidal) pathway through a bi-directional cross talk^11^ (Figure S1). With the exception of HMGR and DXS, expression of all the genes involved in MVA and MEP pathways remained unaffected during phase transitions (Figure 4A,B). However, HMGR and DXS, the initial gene(s) of terpenoid biosynthesis recorded higher expression in LV as compared to LB and LF. Recently, a report in *Medicago truncatula* pointed that stress induced TRITERPENE SAPONIN BIOSYNTHESIS ACTIVATING REGULATOR1 (TSAR1) and TSAR2 (bHLH type) as positive regulators of HMGR governed by physiological and environmental conditions^44^. Differential expression pattern of DXS under strict regulation during different developmental phases was also illustrated in the previous studies^45^. Regulation of these rate limiting steps of terpenoid biosynthesis possibly essential for maintaining the equilibrium between primary and secondary metabolism during developmental phase transitions. The relatively higher expression of KA13H, UGT 85C2, UGT 74G1 and UGT 76G1, during LV supports the concept of optimum SGs accumulation during vegetative phase. While higher expression of GAs-specific genes (KAO, GA20O and GA3O) in LB and LF suggest the shift of ent-kaurenoic acid flux towards GAs synthesis. This could consequently be the potential cause for the reduction in SGs content during the onset of flowering event. Similar observations were also recorded in previous studies in Stevia^14,15,41,46^. Furthermore, the current findings also provide insights into the regulatory mechanism for precise utilization of ent-kaurenoic acid between SGs and GAs biosynthesis. Generally, gibberellins are involved in many processes throughout the plant life-span but their main function has been exclusively studied during shoot apical meristem (SAM) to floral meristem (FM) transition, and altered expression of GAs biosynthesis related genes from vegetative to reproductive phase transitions supports this concept.

Secondary metabolites are involved in plant acclimatization during different stresses and developmental events, therefore, synthesis and accumulation of the bioactive molecules are straightly influenced by both physiological and environmental conditions^47^. The involvement of CYPs and UGTs in the bioconversion and diversification of secondary metabolism has been one of the considerable interests in the recent past^22,24,25^. CYPs (EC 1.14.−.−) are monooxygenases that introduce hydroxyl (–OH) group in a regiospecific manner to provide a modification site for further diversification events^24^. It is evidenced from the current data that a large proportion of Stevia genome was found to be engaged in the synthesis of such proteins. UGTs (EC 2.4.1.−) are glucosyltransferases and perform the integration of activated nucleotide sugar moieties in an acceptor molecule at specific positions to define their bioactivity, solubility and inter and/or intra-cellular transports^22^. Interestingly, 118 transcripts were annotated to encode 45 UGTs involved in several glycosylation processes including SGs biosynthesis.

PPI network analysis became a useful way to identify putative hub proteins, and revealing their relatedness and interactive actions during signaling and regulatory mechanisms^48,49^. Using *Arabidopsis* PPI network, we analyzed the interactive influence of CYPs and UGTs on the SGs/GAs biosynthesis which brought about the higher number of interactions for initial enzymes (AACT, HMGS and DXS) of terpenoid biosynthesis, providing insights about the controlled energy flow between primary and secondary metabolism in Stevia. Interaction of KAO (CYP 88A3), which converts ent-kaurenoic acid into GA12, with three other CYPs and their co-expression during reproductive phase (LB and LF) is an indication of their putative role in GAs biosynthesis. Similarly, the interaction of UGT 85C2 with UGT 92A1 and their co-expressive attributes during vegetative phase (LV) suggest their influence on SGs synthesis. Furthermore, CYPs and UGTs with more expression in LV and significant numbers of neighbours (putative hub proteins) in PPI network were found to be involved in various processes, which directly or indirectly helpful for higher SGs biosynthesis in leaf tissues during vegetative phase. CYP 98A3 was reported to be involved in para- and meta-hydroxylation of cinnamates, which are the part of the chemical defense system to protect vegetative tissues from herbivory attacks^21^. Likewise, CYP 77A1 was found to be involved in anthocyanin synthesis that may require to cope up with different oxidative stresses^50^. These processes are also necessary for proper plant development and therefore, can influence overall SGs content. Contrarily, higher expression of CYP 82C4 and 707A2 during reproductive phase possibly involved in circadian rhythm^51^ and ABA catabolism to reduce its antagonistic action against GA-signaling^52^, respectively. Hence, expression of these CYPs may have a positive influence on vegetative-reproductive phase transition. Constitutive expression of CYP 707A3 irrespective of phase transition (LV, LB, LF), further signifies its role in plant cell metabolism^53^. Furthermore, elevated expression of several UGTs during vegetative phase was known to be involved in cell-cycle regulation and phytohormones signaling, wherein, UGT 92A1 and UGT 74D1 reported to be involved in auxins and cytokinins glycosylation controlling their concentration, and root gravitropism^54,55^. Likewise, UGT 74E2, an H_2_O_2_ induced protein that acts on indole-3-butyric acid (IBA) to maintain auxin homeostasis and signaling, and integrate reactive oxygen species (ROS)^56^. While, UGT 85A2, a membrane-associated protein which express predominately in actively dividing cells^57^ signifies its role in cell cycle regulation.

## Conclusion

In this study, comparative leaf transcriptome demonstrates the advantages of high throughput genomics to accelerate the genome-wide ascertainment of the key gene(s) and regulators for the dissection of complex developmental phase transitions involved in SG biosynthesis in Stevia. Coordinated utilization of ent-kaurenoic acid between SGs and GAs synthesis evident by differential expression and quantitative validations of important genes of MVA and MEP pathway also indicates the presence of a mechanism for homeostatic balance between primary and secondary metabolism. Conclusively, developmental phase dependant expression of many genes (HMGR, DXS, KA13H), transcription factors (MYB, bHLH, WRKY, NAC) and different sets of diversifying enzymes (CYPs and UGTs), can be considered as the putative candidates for manipulating SGs content in Stevia. Further, identified CYPs (124) and UGTs (45) can be the potential targets for plant engineering practices, understanding the evolutionary pattern of secondary metabolism and other important pathways in Stevia. These results represent the first step towards dissection of the complex molecular mechanisms involved in SGs biosynthesis in leaf tissue during developmental phase transition in Stevia. This study provides abundant genomic resources and potential candidates for futuristic studies to upscale SG biosynthesis, and implementation of molecular breeding strategies for genetic improvement of this plant species.

## Materials and Methods

### Plant materials and RNA isolation

Stevia genotypes (CSIR-IHBT-ST-04) were cultivated under long day (16-hr light/8-hr dark) and 60% humidity conditions at 25 °C in growth chamber (Weiss Technk UK Ltd). Considering the growth and developmental period of Stevia (from May-January)^58^, leaves from 1^st^ to 6^th^ successive nodes of three phenotypically healthy plants were harvested at the end of July (LV), in the mid of September (LB), and at the end of October (LF) (Figure 1). Leaves of each node (1^st^ to 6^th^) from three genotypes were pooled and snap-frozen in liquid nitrogen to store at −80 °C. Total RNA was isolated from each pooled leaf sample of three phases using iRIS method^59^. The isolated RNA samples were resolved on 1% denaturing agarose gel to assess their integrity followed by quantification with NanoDrop™ 2000 (Thermo Scientific, Lithuania).

### cDNA library preparation and Illumina sequencing

Equimolar concentration from six RNA samples were pooled for respective phases (LV, LB and LF) and was used for cDNA library preparation. In total, three cDNA libraries were constructed using TruSeq RNA library kit (Illumina, USA) as per the manufacturer’s instructions. Briefly, magnetic beads with Oligo (dT) were used for isolating mRNA, then the purified mRNA was fragmented into shorter fragments and reverse transcribed with Superscript II Reverse transcriptase (Invitrogen, USA) by priming with random hexamers to synthesize the first strand of cDNA. The second strand was synthesized using DNA polymerase I and the overleft single strands were removed by RNase H treatment. The cDNA was cleaned up using Agencourt® AMPure® XP beads (Backman Coulter, USA). Adapters were ligated to the cDNA molecules after end repair and single nucleotide (A) addition followed by washing to remove excess adaptors. The quality of all the libraries was ascertained using Agilent 2100 Bioanalyzer (Agilent Technologies USA) and quantified using Qubit^®^ 2.0 fluorometer (Invitrogen, USA). An equimolar concentration of the three libraries was used for transcriptome sequencing. Finally, the libraries were sequenced on Illumina GAIIx platform following manufacturer’s recommendations to generate 72 bp paired-end reads. Similar sampling and sequencing approach was adopted for generating in-house transcriptome data for young unopened floral bud and full bloomed flower tissues collected during bud phase and flowering phase, respectively.

### *De novo* sequence assembly, validation and functional annotation

After Illumina sequencing, raw reads captured in image form were converted to the readable FASTQ format by base calling method using CASAVA package (ver. 1.8.2). High quality reads were obtained after adaptor removal and quality filtering with default parameters (minimum probability for a read to contain zero errors = 75%, minimum average Phred score for a sequence read = 20, and minimum Phred score for each base of a read = 10) using NGS QC Toolkit^60^. For improving the quality of *de novo* assembly, filtered reads from in-house transcriptome data (unpublished) for young unopened floral bud and full bloomed flower tissues were also used along with the reads obtained from three libraries (LV, LB and LF). CLC Genomics Workbench (ver. 6.5, CLC Bio, Denmark, http://www.clcbio.com) was used to assemble high quality reads with default parameters (trimming quality score = 0.05, similarity fraction = 0.8, mismatch cost = 2, insertion/deletion cost = 3) and a minimum transcript length of 300 bp^28^. Further, to validate the quality of *de novo* assembly, we used two deferent approaches^61^. Firstly, high quality reads were mapped on the assembled transcripts using Bowtie2 tool (ver. 2.2.4)^62^ and secondly, by aligning available EST sequences (https://www.ncbi.nlm.nih.gov/nucest/?term=stevia%20rebaudiana) over assembled transcripts using the BLASTn algorithm.

For functional annotation, *de novo* assembled transcripts were subjected to the BLASTx algorithm (e-value cut off of ≤1e^−5^)^63^ against different databases such as NCBI’s nr, Swiss-Prot (http://www.expasy.ch/sprot), TAIR 10, and PTF database ver. 3.0 (http://planttfdb.cbi.pku.edu.cn/) to retrieve the top hits showing highest sequence similarity. The transcripts having homologs in TAIR10 database were assigned specific GO terms to classify them into three broad categories (biological processes, molecular functions and cellular components) using AgriGO toolkit^64^. To identify and characterize the active metabolic pathways in Stevia, the KEGG database (http://www.genome.jp/kegg) was used. Identified enzymes were assigned their respective enzyme commission (EC) numbers and further classified into six major classes namely, Oxidoreductases, Transferases, Hydrolases, Lyases, Isomerases and Ligases. Furthermore, CYPs and UGTs, the putative key candidates for diversification of plant metabolism during developmental phase transition^21^ were identified from Swiss-Prot (http://www.expasy.ch/sprot) annotation followed by their classification in respective families.

### Statistical analysis and identification of differentially expressed genes

To compute the transcript abundance, the filtered reads of three libraries (LV, LB and LF) were aligned individually to *de novo* assembled transcripts using Bowtie2 tool (ver. 2.2.4)^60^. The expression level of each transcript was measured in terms of RPKM^65^. edgeR, a Bioconductor package based on negative binomial distribution^66^, was used to identify differentially expressed genes in the pair-wise comparative analysis of different leaf tissues with false discovery rate (FDR) < 0.05 and log2 fold change ≥2.

### Quantitative polymerase chain reaction (qRT-PCR) validation

To support the efficacy of gene expression in RNA-Seq analysis, key genes of SGs and GAs biosynthesis were selected for qRT-PCR validation. Gene specific primers were designed using BatchPrimer3 (http://probes.pw.usda.gov/batchprimer3/) and their related information is listed in Table S9. Total RNA was isolated from leaf tissues of respective phases (LV, LB and LF) followed by removal of genomic DNA contamination using DNase I (Thermo Scientific, Lithuania) treatment. 2 µg of purified RNA was used for reverse transcription to prepare cDNA using RevertAid H Minus First Strand cDNA Synthesis Kit (Thermo Scientific, Lithuania). qRT-PCR was performed with a StepOnePlus™ Real-Time PCR System (Applied Biosystems, USA) in a 20 µl reaction volume containing 200 ng cDNA, Power SYBR® Green PCR Master Mix (Applied Biosystems, USA) and gene-specific primers. GAPDH was used as an internal control to maintain the equality of template in all reactions. Expression analysis of all the genes was performed in triplicate and relative gene expression was calculated by applying 2^−ΔΔCt^ method^67^.

### PPI network analysis

Further, to understand the impact and interaction of CYPs and UGTs with proteins involved in SGs biosynthesis, the TAIR annotations of all CYPs, UGTs, and genes involved in SGs and GAs biosynthesis were used for PPI network analysis. For this, a predetermined PPI network of *Arabidopsis thaliana* (AtPIN, http://ftp.arabidopsis.org/home/tair/Proteins/Protein_interaction_data/Interactome2.0/)^68^, was used as a template due to lack of reference genome of Stevia. Cytoscape software (version 2.8)^28^ was used for visualization of PPI network and identification of crucial modules (putative master regulators) after considering the first neighbour of mapped TAIR IDs. It is suggested that if two selected Stevia proteins corresponded to two homologous proteins in the template *Arabidopsis* network, the encoded proteins were also considered to interact with each other in predicted Stevia network^69,70^. The degree of the predicted network was defined as the number of neighbors of each node to identify putative hub proteins^49^. Further, the integration of protein interactions and mRNA expression profiles of selected genes were analysed^17,49^ to predict their putative role in different metabolic processes during developmental phase transitions.

## Electronic supplementary material

Supplementary information

Table S2

Table S3

Table S4

Table S5

Table S6

Table S7

Table S9
