# Supplementary material for: Molecular dissection of transcriptional reprogramming of steviol glycosides synthesis in leaf tissue during developmental phase transitions in Stevia rebaudiana Bert
Source: Sci Rep. 2017 Sep 19;7:11835. doi: 10.1038/s41598-017-12025-y (PMC5605536; doi:10.1038/s41598-017-12025-y)
Supplement: Supplementary file 1 — Supplementary information [file 41598_2017_12025_MOESM1_ESM.pdf]

Supplementary Information

Revised Original Research Article SREP-17-10793

Molecular dissection of transcriptional reprogramming of steviol glycosides synthesis in leaf tissue during developmental phase transitions in *Stevia rebaudiana* Bert

Gopal Singh[1,2], Gagandeep Singh[1], Pradeep Singh[1], Rajni Parmar[1,2], Navgeet Paul[1], Radhika Vashist[1], Mohit Kumar Swarnkar[1], Ashok Kumar[3], Sanatsujat Singh[3], Anil Kumar Singh[1†], Sanjay Kumar[1], Ram Kumar Sharma[1,2]*

[1]Biotechnology Department, CSIR-Institute of Himalayan Bioresource Technology, Palampur, Himachal Pradesh, India

[2]Academy of Scientific and Innovative Research, New Delhi, India

[3]Agrotechnology of Medicinal, Aromatic and Commercially Important Plants, CSIR-Institute of Himalayan Bioresource Technology, Palampur, Himachal Pradesh, India

†Present address: ICAR-Indian Institute of Agricultural Biotechnology, PDU Campus, IINRG, Namkum, Ranchi, Jharkhand, India

***Correspondence:**

Dr Ram Kumar Sharma
Biotechnology Division
CSIR-Institute of Himalayan Bioresource Technology
Palampur (H.P)
India
Email: rksharma.ihbt@gmail.com, ramsharma@ihbt.res.in

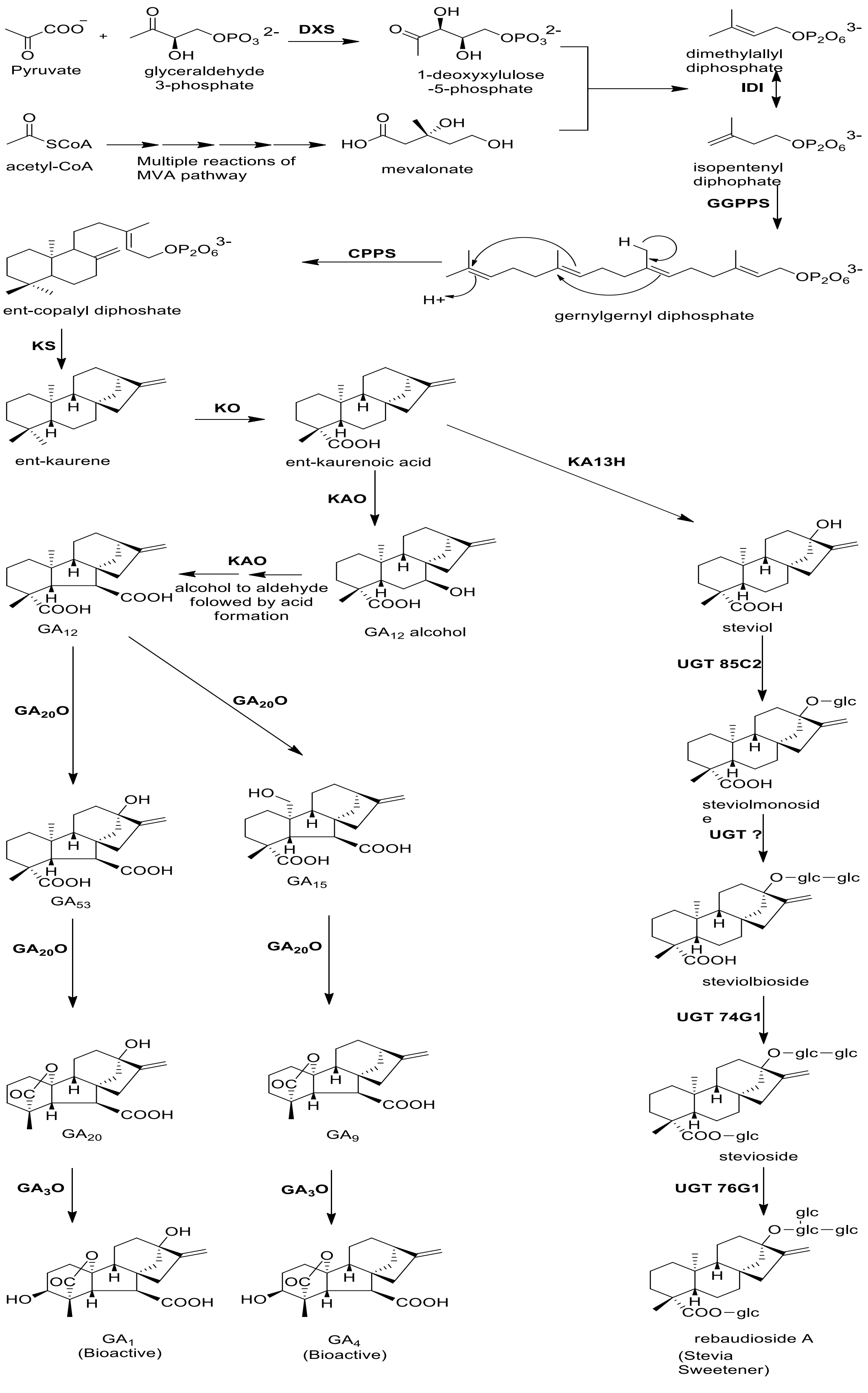
Pyruvate
glyceraldehyde 3-phosphate
DXS
1-deoxyxylulose -5-phosphate
dimethylallyl diphosphate
IDI
acetyl-CoA
Multiple reactions of MVA pathway
mevalonate
isopentenyl diphophate
GGPPS
CPPS
ent-copalyl diphoshate
gernylgernyl diphosphate
KS
ent-kaurene
KO
ent-kaurenoic acid
KA13H
KAO
steviol
KAO
alcohol to aldehyde folowed by acid formation
GA12 alcohol
GA12
UGT 85C2
GA20O
GA20O
steviolmonoside
UGT ?
GA53
GA15
steviolbioside
GA20O
GA20O
UGT 74G1
GA20
GA9
stevioside
GA3O
GA3O
UGT 76G1
GA1 (Bioactive)
GA4 (Bioactive)
rebaudioside A (Stevia Sweetener)

Figure S1

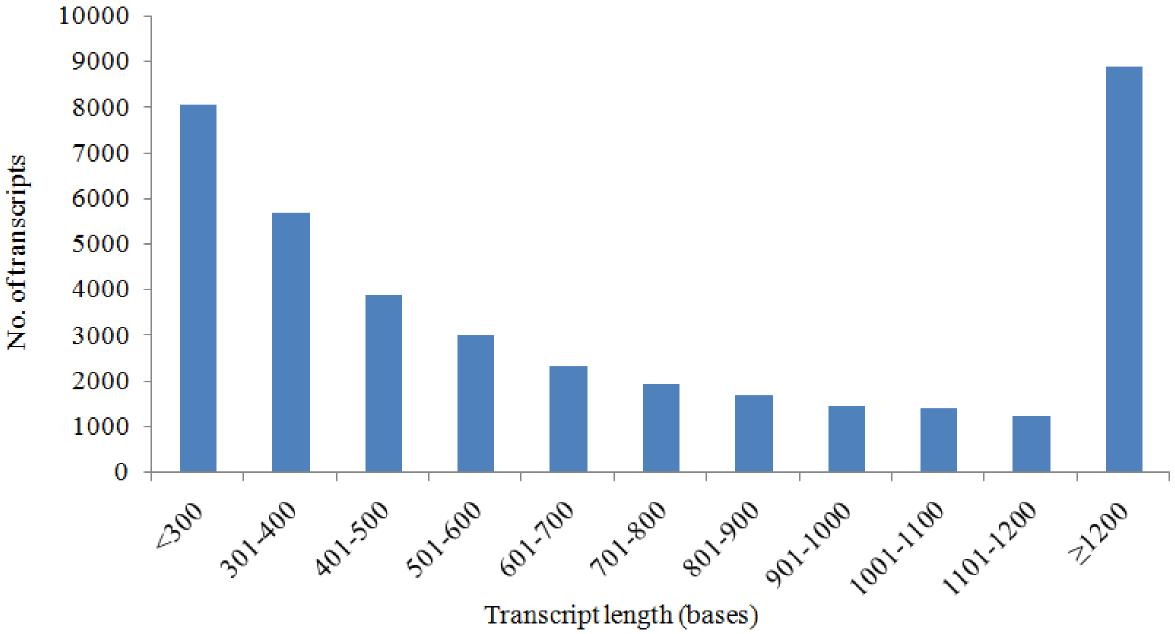
10000
9000
8000
7000
6000
5000
4000
3000
2000
1000
0
No. of transcripts
<300
301-400
401-500
501-600
601-700
701-800
801-900
901-1000
1001-1100
1101-1200
≥1200
Transcript length (bases)

Figure S2

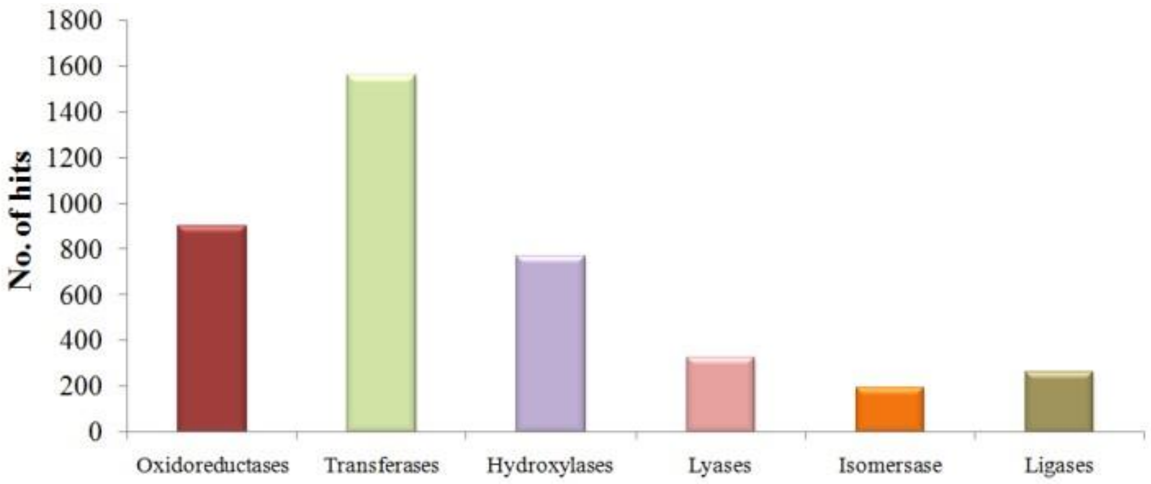
1800
1600
1400
1200
1000
800
600
400
200
0
No. of hits
Oxidoreductases
Transferases
Hydroxylases
Lyases
Isomersase
Ligases

Figure S3

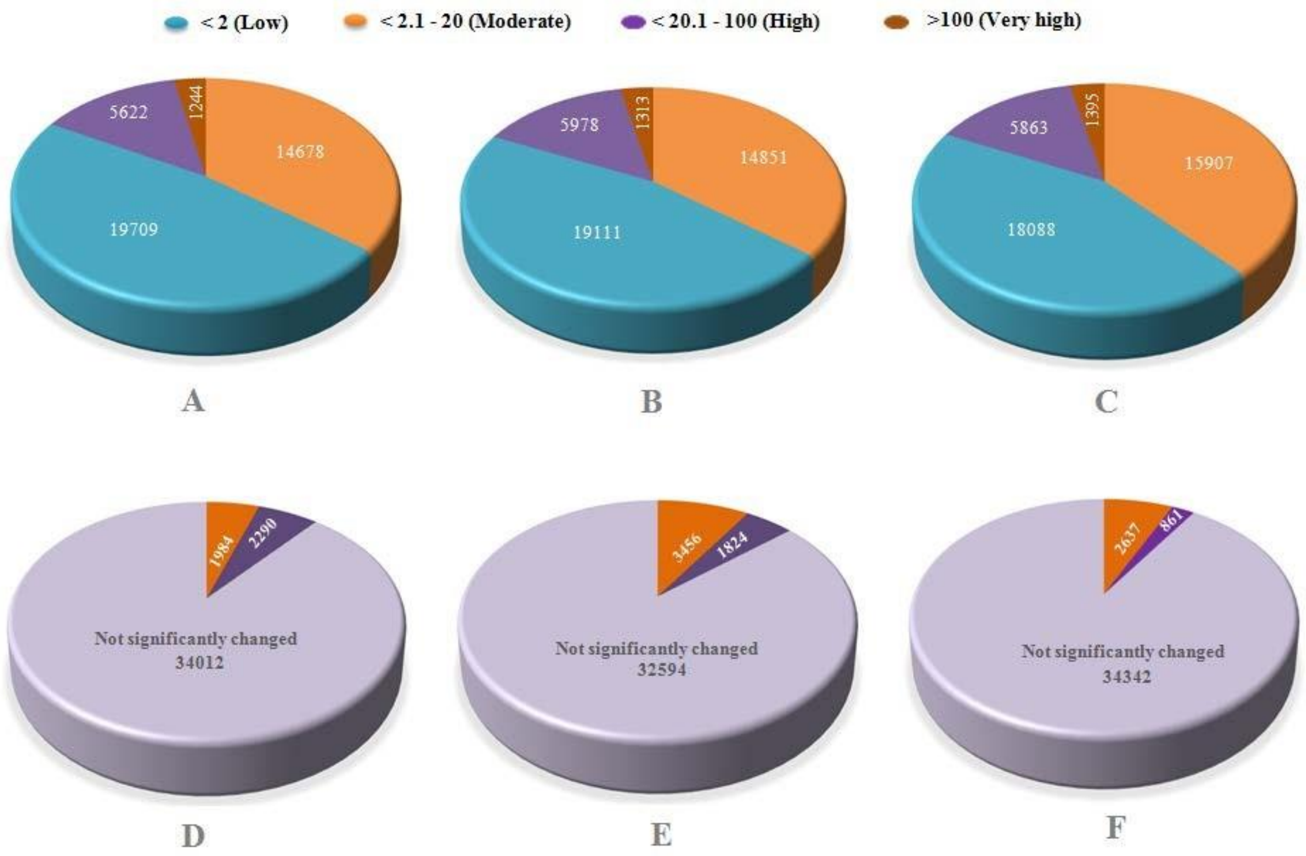
< 2 (Low)
< 2.1 - 20 (Moderate)
< 20.1 - 100 (High)
>100 (Very high)
5622
1244
14678
19709
A
5978
1313
14851
19111
B
5863
1395
15907
18088
C
1984
2290
Not significantly changed
34012
D
3456
1824
Not significantly changed
32594
E
2637
861
Not significantly changed
34342
F

Figure S4

Table S1 Summery of *de novo* assembly obtained with CLC Genomics Workbench for Stevia transcriptome analysis.

Total reads	82,293,565
Total reads length (bases)	5,925,136,680
Total matched reads	66,264,953
Total length of matched reads (bases)	4,771,076,616
Percent of matched reads (%)	80.53
Total unmatched reads	16,028,612
Total length of unmatched reads (bases)	1,154,060,064
Percent of unmatched reads (%)	19.47
Total assembled transcripts	41,262
Total length of transcripts (bases)	38,034,545
N75 (bases)	670
N50 (bases)	1,244
N25 (bases)	1,969
Longest transcript (bases)	12,230
Shortest length (bases)	269
Average length (bases)	922
GC %	39.3

Table S8 List of CYPs and UGTs with their respective TAIR IDs having ≥5 neighbours in PPI network analysis

Transcripts	Protein name	TAIR ID	TAIR annotation	Degree	TAIR IDs of first neighbours
stevia5_contig_752	CYP 81D3	AT4G37340	CytochromeP450, family 81, subfamilyD, polypeptide3	13	AT3G26310 AT3G26330AT4G01030 AT4G37430AT3G26200 AT3G26320 AT2G02580AT2G37320 AT1G06580AT3G26300 AT5G10610 AT4G37370AT3G26290
stevia5_contig_5166	UGT74E2	AT1G05680	Uridinediphosphate glycosyltransferase74E2	6	AT2G23210 AT5G54010AT1G30530 AT5G59580AT5G38010 AT4G27570
stevia5_contig_2344	CYP 72A15	AT3G14690	CytochromeP450, family 72, subfamilyA, polypeptide15	14	AT2G45560 AT3G14620AT5G04660 AT1G73340AT3G14640 AT1G67110 AT3G14680AT3G14660 AT3G14610AT5G04330 AT4G31950 AT3G14650AT2G12190 AT2G45570
stevia5_contig_1958	CYP 98A3	AT2G40890	CytochromeP450, family 98, subfamilyA, polypeptide3	5	AT4G39510 AT5G57740AT1G74540 AT3G14630AT3G04130
stevia5_contig_4046	CYP 704A2	AT2G45510	CytochromeP450, family 704, subfamilyA, polypeptide2	7	AT1G33720 AT1G64940AT3G03470 AT1G64930AT1G73720 AT2G45550 AT5G47990
stevia5_contig_6547	UGT92A1	AT5G12890	UDP-Glycosyltransferase superfamilyprotein	5	AT2G36770 AT1G22380AT2G36760 AT4G34138AT3G55580
stevia5_contig_2692	CYP 77A1	AT5G04660	CytochromeP450, family 77, subfamilyA, polypeptide4	12	AT2G45560 AT1G73340AT1G67110 AT3G14680AT3G14660 AT3G14690 AT3G14610AT5G04330 AT4G31950AT3G14650 AT2G12190 AT2G45570
stevia5_contig_23361	UGT74D1	AT2G31750	UDP-glucosyltransferase 74D1	6	AT2G31790 AT2G23260AT3G02100 AT4G14090AT2G28840 AT1G24100
stevia5_contig_5356	CYP 82G1	AT3G25180	CytochromeP450, family 82, subfamilyG, polypeptide1	7	AT2G23220 AT4G31970AT3G14620 AT3G20130AT4G31950 AT2G25160 AT2G45580
stevia5_contig_1578	CYP 71A21	AT3G48320	CytochromeP450, family 71, subfamilyA, polypeptide21	10	AT5G35917 AT4G13290AT1G05160 AT3G48290AT1G13090 AT3G48270 AT1G13100AT3G48280 AT3G26830AT3G48310
stevia5_contig_13802	CYP 706A3	AT5G44620	CytochromeP450, family 706, subfamilyA, polypeptide3	5	AT2G46960 AT5G52400AT4G39490 AT1G13140AT1G50560
stevia5_contig_904	CYP 75B1	AT5G07990	CytochromeP450 superfamilyprotein	6	AT5G58860 AT4G12320AT3G10570 AT3G20100AT3G50660 AT4G12300

stevia5_contig_760	UGT85A2	AT1G22360	UDP-glucosyltransferase 85A2	8	AT5G26310 AT2G29750AT1G05560 AT1G22340AT5G66690 AT4G34131 AT1G22370AT1G01420
stevia5_contig_5177	CYP 72A13	AT3G14660	CytochromeP450, family 72, subfamilyA, polypeptide13	9	AT5G04660 AT1G73340AT1G67110 AT3G14680AT3G14690 AT5G04330 AT4G31950AT3G14650 AT2G12190
stevia5_contig_4794	CYP 71A24	AT3G48290	CytochromeP450, family 71, subfamilyA, polypeptide24	6	AT3G48320 AT1G11680AT5G08250 AT3G48270AT3G15430 AT3G55580
stevia5_contig_14900	CYP 72A8	AT3G14620	CytochromeP450, family 72, subfamilyA, polypeptide8	8	AT2G23220 AT3G14640AT3G25180 AT3G20130AT3G14690 AT3G14610 AT3G14650AT2G45580
stevia5_contig_4381	CYP 71A22	AT3G48310	CytochromeP450, family 71, subfamilyA, polypeptide22	8	AT5G35917 AT4G13290AT3G48320 AT1G05160AT1G13090 AT1G13100 AT3G48280AT3G26830
stevia5_contig_15794	CYP 706A7	AT4G12330	CytochromeP450, family 706, subfamilyA, polypeptide7	5	AT2G14100 AT4G27710AT4G37310 AT5G48000AT5G38450
stevia5_contig_13404	CYP 71B35	AT3G26310	CytochromeP450, family 71, subfamilyB, polypeptide35	12	AT3G26330 AT4G01030AT4G37430 AT3G26200AT4G37340 AT3G26320 AT2G02580AT2G37320 AT1G06580AT3G26300 AT5G10610 AT3G26290
stevia5_contig_6041	CYP 89A5	AT1G64950	CytochromeP450, family 89, subfamilyA, polypeptide5	9	AT3G20140 AT5G61320AT3G20110 AT1G11600AT1G64900 AT1G64930 AT2G21910AT1G01280 AT5G63450
stevia5_contig_3177	CYP 72A14	AT3G14680	CytochromeP450, family 72, subfamilyA, polypeptide14	13	AT5G04660 AT1G73340AT1G67110 AT3G14630AT3G14660 AT3G14690 AT3G14610AT1G17060 AT5G04330AT4G31950 AT3G14650 AT2G38280AT2G12190
stevia5_contig_3090	UGT73C4	AT2G36770	UDP-Glycosyltransferase superfamilyprotein	5	AT3G21560 AT2G36780AT5G23575 AT2G36760AT5G12890
stevia5_contig_2012	UGT83A1	AT3G02100	UDP-Glycosyltransferase superfamilyprotein	5	AT2G31790 AT2G31750AT3G55710 AT5G05870AT1G24100

Supplementary Information

Supplementary figures

Figure S1 Overview of biochemical conversion in SGs and GAs biosynthesis. Abbreviations are as follows: DXS (1-deoxy-D-xylulose-5-phosphate synthase), IDI (isopentenyl-diphosphate delta-isomerase), GGPPS (geranylgeranyl pyrophosphate synthase), CPPS (ent-copalylpyrophosphate synthase),KS (ent-copalyl diphosphate synthase), KO (ent-kaurene oxidase), KA13H (ent-kaurenoic acid 13-hydroxylase), UGT 85C2 (UDP-glycosyltransferase 85C2), UGT 74G1 (UDP-glycosyltransferase 74G1), UGT 76G1 (UDP-glycosyltransferase 76G1),UGT ? (unknown UGT), KAO (ent-kaurenoic acid oxidase), GA 20-O (gibberellin 20 oxidase), and GA 3-O (gibberellin 3 oxidase).

Figure S2 Histogram representing the transcripts length distribution in Stevia transcriptome analysis.

Figure S3 Details of enzyme classification obtained in KEGG annotations.

Figure S4 Comparative gene expressions in leaf tissues during development phase transitions in vegetative (LV), budding (LB) and flowering (LF) based on RPKM distribution and edger analysis. Expression level (low, moderate, high and very high expression categories in LV(A), LB(B) and LF(C); Pair-wise comparative differential gene expression of LV *vs* LB(D), LV *vs* LF(E) and LB *vs* LF (F) in edgeR (transcripts showing ≥log2 fold change and FDR <0.05were considered up-regulated).

Supplementary tables

Table S1 Summery of *de novo* assembly obtained with CLC Genomics Workbench for Stevia transcriptome analysis.

Table S2 Summarization of all KEGG hits with their respective metabolic pathways and physiological processes **(Submitted as Supplementary dataset)**

Table S3 Identification and categorization of cytochrome P450-monooxygenases (CYPs) in Swiss-prot annotations **(Submitted as Supplementary dataset)**.

Table S4 Identification and categorization of UDP-glycosyltransferases (UGTs) in Swiss-prot annotations **(Submitted as Supplementary dataset)**.

Table S5 Comparative differential gene expression analysis of assembled transcripts based on edger statistics along with their respective NCBI's nr annotations **(Submitted as Supplementary dataset)**.

Table S6 Identification and functional annotations for all the genes of SGs and GAs synthesis with different databases **(Submitted as Supplementary dataset)**.

Table S7 Summary of protein-protein interactome network representing TAIR IDs of interacting proteins with annotations followed by degrees and TAIR IDs of their neighbours **(Submitted as Supplementary dataset)**

Table S8 List of CYPs and UGTs with their respective TAIR IDs having ≥5neighbours in PPI network analysis.

Table S9 List of primer sequences of selected transcripts used for qRT-PCR **(Submitted as Supplementary dataset)**.
